# BARRIERS AND FACILITATORS TO HELP-SEEKING FOR ELDER ABUSE: A QUALITATIVE STUDY FROM THE PERSPECTIVE OF VICTIMS

**DOI:** 10.1093/geroni/igae098.2065

**Published:** 2024-12-31

**Authors:** Andie MacNeil, Jessica Hsieh, Isabel Rollandi, Jo Anne Sirey, Mark Lachs, David Burnes

**Affiliations:** University of Toronto, Toronto, Ontario, Canada; University of Toronto, Toronto, Ontario, Canada; Weill Cornell Medicine, New York, New York, United States; Weill Cornell Medicine, New York, New York, United States; Weill Cornell Medicine, New York, New York, United States; University of Toronto, Toronto, Ontario, Canada

## Abstract

Understanding help-seeking among victims of elder abuse (EA) is a major challenge and significant knowledge gap in the field. The vast majority of EA victims are not connected with formal response systems, such as Adult Protective Services, law enforcement, and other agencies responsible for addressing this issue in the community. The current study sought to understand barriers and facilitators to help-seeking from the perspective of EA victims. Using a descriptive phenomenological approach, this study conducted in-depth, individual, qualitative interviews with a diverse sample (n = 32) of EA victims living in New York City and Los Angeles, United States. Analysis followed a constant comparison process involving two independent coders of verbatim transcripts. Major themes that emerged as barriers to help-seeking included: embarrassment or shame, self-blame or guilt, fear of perpetrator retaliation, fear of what could happen to perpetrator, concern for family preservation, and lack of awareness of services. Facilitators in the process of help-seeking included: reaching a “tipping point”, concern for self-preservation, accessibility and convenience of services, and having knowledge about where and how to seek support. These findings emphasize that barriers and facilitators to help-seeking span various levels of ecological influence, including the individual victim, the individual perpetrator, the victim-perpetrator relationship, and the family system. To promote greater help-seeking among EA victims, there must be accessible interventions that help address the complex and interrelated psychological, emotional, relational, and familial factors that impact help-seeking behavior among EA victims.

